# Multi‐omics analysis of the oncogenic value of copper Metabolism‐Related protein COMMD2 in human cancers

**DOI:** 10.1002/cam4.5320

**Published:** 2022-10-07

**Authors:** Panpan Tai, Zhanwang Wang, Xinyu Chen, Aiyan Chen, Lian Gong, Yaxin Cheng, Ke Cao

**Affiliations:** ^1^ Department of Oncology Third Xiangya Hospital of Central South University Changsha China

**Keywords:** bioinformatics, COMMD2, immune infiltration, pan‐cancer, prognostic biomarker

## Abstract

**Background:**

The copper metabolism MURR1 domain (COMMD) protein family is involved in tumorigenicity of malignant tumors. However, as the member of COMMD, the role of COMMD2 in human tumors remains unknown.

**Methods:**

We used The Cancer Genome Atlas (TCGA), Genotype Tissue Expression (GTEx), Human Protein Atlas (HPA) database, Cancer Cell Line Encyclopedia (CCLE) platform, univariate Cox regression analysis, Kaplan–Meier curve, cBioPortal, UALCAN database, Sangerbox online platform, GSCA database gene set enrichment analysis (GSEA), and GeneMANIA to analyze the expression of COMMD2, its prognostic values, genomic alteration patterns, and the correlation with tumor stemness, tumor mutational burden (TMB), microsatellite instability (MSI), and immune infiltrates, drug sensitivity, and gene function enrichment in pan‐cancer. qRT‐PCR, CCK‐8, EdU, wound healing, and transwell migration assays were performed to confirm the function of COMMD2.

**Results:**

COMMD2 was strongly expressed in most cancer types. Elevated COMMD2 expression affects the prognosis, clinicopathological stage, and molecular or immune subtypes of various tumors. Moreover, promoter hypomethylation and mutations in the COMMD2 gene may be associated with its high expression and poor survival. Additionally, we discovered that COMMD2 expression was linked to tumor stemness, TMB, MSI, immune cell infiltration, immune‐checkpoint inhibitors, and drug sensitivity in pan‐cancer. Furthermore, the COMMD2 gene co‐expression network is constructed with GSEA analysis, displaying significant interaction of COMMD2 with E2F targets, G2‐M checkpoint, and mitotic spindle in bladder cancer (BLCA). Finally, RNA interference data showed suppression of COMMD2 prevented proliferation and migration of BLCA and uterine corpus endometrial carcinoma (UCEC) cells.

**Conclusion:**

Our findings shed light on the COMMD2 functions in human cancers and demonstrate that it is a promising prognostic biomarker and therapeutic target in pan‐cancer.

## Graphical abstract

The oncogenic value of COMMD2 in human cancers.

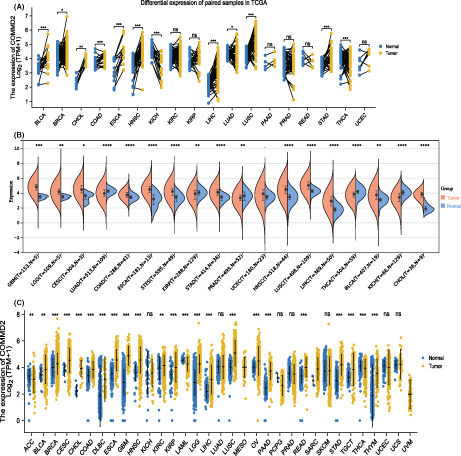

## 1 INTRODUCTION

Cancer is anticipated to be the top cause of mortality and the single most significant challenge to improving the life span across every country on the planet.^1^ To improve patient survival, it is essential to identify novel diagnostic and prognostic biomarkers as well as therapeutic targets. The advancement of a large number of public databases, such as The Cancer Genome Atlas (TCGA) and Gene Expression Omnibus (GEO), has enabled researchers to investigate beneficial aspects.^2^

The copper metabolism MURR1 domain (COMMD) proteins is a conserved family which plays important roles in biological function,^3^, ^4^ including copper metabolism,^5^, ^6^ blood‐lipid regulation,^7^ Na^+^ homeostasis,^8^ NF‐κB pathway,^9^, ^10^ and membrane protein transport.^11^ Proteins of the COMMD family are intimately involved in human malignancies. COMMD1 was found to negatively regulate NF‐κB by controlling the ubiquitination of NF‐κB components and interrupting the dimerization of HIF‐1 alpha/beta to inhibit human cancers.^9^, ^12^, ^13^ COMMD3 has been found to be associated with the initiation and development of metastatic prostate and liver malignancies.^14^, ^15^ In non‐small cell lung cancer (NSCLC) cells, COMMD4 expression is increased, and siRNA‐mediated COMMD4 knockdown reduces cell proliferation, migration, and viability.^16^ Furthermore, COMMD5/HCaRG is engaged in tissue repair, and its weak expression has been correlated with tumorigenicity.^17^ By altering the expression or phosphorylation of ErbB members, COMMD5 suppresses the growth of renal cancer cells.^18^ NF‐κB activation could be entirely eliminated by altering the amino acid residues of Trp24 and Pro41 in the COMMD domain of COMMD6,^19^ indicating its underlying tumor promoting function in head and neck squamous cell carcinoma and cholangiocarcinoma.^20^ Furthermore, silencing of COMMD7 inhibits hepatocellular carcinoma (HCC) cell proliferation, migration, and invasion by suppressing NF‐κB p65,^21^ thereby acting as a new molecular target in the late stages of pancreatic ductal adenocarcinoma.^22^ In vitro silencing of COMMD8 blocked NSCLC cell proliferation, colony formation, and glycolysis along with acceleration of apoptosis.^23^, ^24^ siRNA‐induced suppression of COMMD9 diminished TFDP1/E2F1 activation, proliferation, and migration; stopped the cell cycle at G1/S transition; and induced autophagy in NSCLC cells.^25^ Moreover, in HCC, COMMD10 suppresses TNF‐mediated ubiquitination of IκBα and p65 nuclear translocation via the coupling of the COMMD10‐N terminal to the Rel homology domain of p65, along with increase of NF‐κB inactivity and production of cleaved caspase 9/3, thereby hampering the progression of HCC.^26^

Although the COMMD family has been studied in human cancers, the expression and role of COMMD2 in tumors remains unknown. The purpose of this study was to discover the expression of COMMD2 and its prognostic significance in pan‐cancer. The correlation between COMMD2 expression and immune and molecular subtypes of various cancers has been studied. Furthermore, we investigated the relationships between COMMD2 expression and genomic alterations, TMB, MSI, and immune cell infiltrates in human cancers. COMMD2's pro‐cancer activities in bladder cancer cells and uterine corpus endometrial carcinoma cells have also been explored. In conclusion, our findings add to our understanding of COMMD2's carcinogenic role, which could be employed as a prognostic biomarker and therapeutic target in pan‐cancer.

## 2 MATERIALS AND METHODS

### 2.1 Clinical data acquisition and expression of COMMD2

Pan‐cancer transcriptional RNA sequencing and clinical data were obtained from The Cancer Genome Atlas (TCGA) and Genotype‐Tissue Expression (GTEx). FPKM expression data were transformed to the TPM value and transformed by log2.^27^ The analysis of COMMD2 expression in cell lines was based on CCLE (https://sites.broad institute.org/ccle), while the SangerBox database (http://www.sangerbox.com/) was used to identify the differential expression of COMMD2. The Wilcoxon rank‐ sum test was done for unpaired samples, and paired test was performed in case of paired tissue samples, *p*‐value <0.05 considered a significant standard.

### 2.2 Protein level analysis of COMMD2

To explore the differential expression of COMMD2 at the protein expression level, immuno‐ histochemical analysis was performed on eight different types of tumor tissue samples along with corresponding normal tissues from HPA (https://www.protein atlas.org/), including liver hepatocellular carcinoma (LIHC), BLCA, testicular germ cell tumors (TGCT), ovarian cancer (OV), prostate cancer (PRAD), pancreatic cancer (PAAD), glioblastoma (GBM), and head and neck cancer (HNSC).

### 2.3 Survival analysis of COMMD2

To mine the relationship between COMMD2 gene expression and patient prognosis, univariate Cox regression analysis and Kaplan–Meier curves were performed to examine patients' overall survival (OS) and disease‐specific survival (DSS) in pan‐cancer using the TCGA database.

### 2.4 Clinicopathological features associated with COMMD2 expression

The TISIDB database (http://cis.hku.hk/TISIDB/) is a user‐friendly integrated repository portal for tumor–immune system interactions.^28^ The association of COMMD2 expression with the stage, grade, and molecular or immune subtypes of various tumors was probed using the TISIDB database.

### 2.5 Analysis of methylation and genetic alternations of COMMD2

The UALCAN database (http://ualcan.path.uab.edu/analysis.html) was utilized to investigate the differences in methylation of COMMD2 between normal and tumor samples. Genetic alterations were evaluated using cBioPortal (http://www.cbioportal.org/) on the basis of the TCGA Pan‐Cancer Atlas Studies data.

### 2.6 Relationship of COMMD2 with tumor stemness index, tumor mutational burden, and microsatellite instability

The tumor stemness index reflects the degree of tumor differentiation. mRNAsi is based on gene expression, whereas mDNAsi is based on the level of DNA methylation. The relationship between COMMD2 gene expression and the tumor stemness index, tumor mutational burden (TMB), and microsatellite instability (MSI) was determined using the SangerBox database (http://www.sangerbox.com/).

### 2.7 COMMD2 expression and immunity

The TIMER, MCPCOUNTER, and QUANTISEQ algorithms were employed to evaluate the relationship between COMMD2 expression and multiple immune cell infiltration in different tumors, and we calculated the immuneScore, stromalScore, and ESTIMATEScore using the SangerBox database (http://www.sangerbox.com/).^29^

### 2.8 Association of COMMD2 expression with immune checkpoint inhibitors and drug sensitivity

The correlation between COMMD2 mRNA expression and immune checkpoint inhibitors and treatment response‐related biomarkers relies on the TCGA data. GSCA (http://bioinfo.life.hust.edu.cn/GSCA/#/) is an online platform that integrates multi‐ omics data based on the TCGA database. The association between COMMD2 gene expression and drug sensitivity was analyzed using the GSCA database.

### 2.9 Gene–gene interaction and enrichment analysis of COMMD2

The GeneMANIA database (http://www.genemania.org) is a handy tool that can build a gene–gene interaction network and aggregate function‐similar genes.^30^ A gene–gene interaction network was established applying the GeneMANIA online platform. To further explore the significant biological role of COMMD2 in human tumors, LinkedOmics (http://www.linkedomics.org/login.php) was conducted to identify the differentially expressed genes related to COMMD2 in the BLCA (*p‐*value <0.05), while gene set enrichment analysis (GSEA) was performed with the gene set. “c5.bp.v7.2. symbols.gmt” and “h.all.v7.2. symbols.gmt” were used as the references. Subsequently, the relevance of COMMD2 expression and activity of various signaling pathways in BLCA were investigated.

### 2.10 Cell Culture and Transfection

BLCA cell lines (UMUC3) and UCEC cell lines (HEC‐1‐A) were purchased from ATCC (Rockville, MD, USA), and cultured in DMEM (Invitrogen, New York, United States) containing 10% FBS (FBSPl; Gibco) and 1% penicillin–streptomycin at 37°C and 5% CO_2_. siRNA and negative controls were purchased from GenePharma (Suzhou, China). The sequences of siRNAs targeting COMMD2 (termed si‐C2#1, si‐C2#2, si‐C2#3) are shown in Table S1.

### 2.11 Quantitative real‐time PCR (qRT‐PCR)

Total RNA from the cultured cells was extracted using Fastern reagent (Invitrogen) according to the manufacturer's instructions. 1 μg of total RNA was reverse‐transcribed into cDNA using the PrimeScript RT Reagent Kit (TaKaRa), and SYBR Green PCR Master Mix was used for qRT‐PCR. The primers were synthesized by Tsingke Biotechnology Co., Ltd. The relative RNA expression level was determined using the 2^−△△CT^ method with GAPDH as an internal loading control. The primer sequences used are presented in Table S1.

### 2.12 Cell Counting Kit‐8 Assay

UMUC3 and HEC‐1‐A cells were transferred into 96‐well plates at a seeding density of 3500 and 6000 cells/well (100 μl medium) and cultured for the indicated time points. 10 μl of the Cell Counting Kit‐8 (CCK‐8) reagent (Biosharp Life Sciences) were added to each well and then the cells were incubated with CCK‐8 reagent for 1.5 hours. The optical density (OD) was measured through a microplate reader at 450 nm.

### 2.13 EdU assay

EdU was diluted to 10 μmol/L using complete medium, and then 100 μl was added to each well and incubated for 4 h, as per kit instructions. Following incubation, the medium was removed, and the cells were fixed with paraformaldehyde after rinsing twice with PBS. Cell multiplication was then identified under a fluorescence microscope using Apollo staining.

### 2.14 Colony formation assay

After transfection for 24 h, the UMUC3 and HEC‐1‐A cells were counted and cell concentration adjusted to 1000 cells/well in 6‐well plates. After incubation for 2–3 weeks, cell colony formation was detected.

### 2.15 Wound‐healing assay

Cells were transfected after the appropriate time point and plated in 6‐well plates. The cells were scratched with sterile 10‐μl pipette tips until they fused to a 100% confluent cell monolayer. Then, serum‐free medium was added to the plates, and images were acquired at 0 h and 24 h to analyze the wound healing distance.

### 2.16 Transwell assay

After 24 h, the cells were collected and cleaned with PBS and serum‐free DMEM. Following this, 200ul of serum‐free cell suspension was added to the upper chamber, and 600ul of DMEM containing 20% FBS was placed in the lower chamber. The chambers were incubated for 24 h. Finally, the cells that had migrated across the membrane were counted and imaged using a light microscope after fixing and staining with crystal violet.

### 2.17 Immunohistochemical Staining

From January 2021 to April 2022, 10 formalin‐fixed, paraffin‐embedded BLCA and UCEC and paired normal bladder and uterine corpus endometrial tissues were collected in The Third Xiangya Hospital of Central South University. In brief, first, the paraffin‐embedded tissue sections were rehydrated and then the endogenous peroxidase activity was blocked by 3% hydrogen peroxide for 25 min at 25°C. After being incubated with COMMD2 primary antibody (Abcam, 1:200) at 4°C overnight, the sections were incubated by the secondary antibody for 90 min at room temperature. Finally, images were collected with a light microscope. The procedures were approved by the Ethics Committee of Third Xiangya Hospital (Changsha, Hunan, China).

### 2.18 Statistical analysis

Gene expression data were converted by log2 normalization. Differential expression analysis in tumors and normal samples was performed using “t‐tests.” For survival analyses, KM analyses, Cox proportional hazard model, and log‐rank test were conducted. Correlations between COMMD2 expression and TMB, MSI, mRNAsi, and mDNAsi were analyzed using Spearman's method. Spearman's or Pearson's correlation coefficient was used to affirm the interactions between variables. Statistical threshold was set to *p*‐value <0.05, or *p*‐adjust <0.05 in our analysis.

## 3 RESULTS

### 3.1 Differential expression profile of COMMD2 in pan‐cancer

**FIGURE 1:**
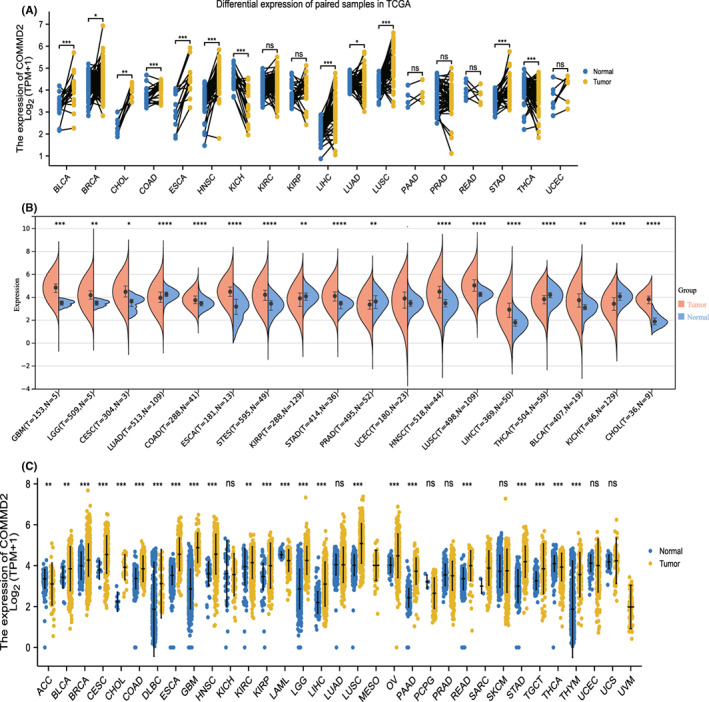
Expression of COMMD2 in tumor and normal tissue. (A) Differential expression of COMMD2 between tumor tissues and paired non‐tumor tissues in the TCGA database. (B) Differential expression of COMMD2 in tumor and non‐tumor tissues in the SangerBox database. (C) Differential expression of COMMD2 in TCGA combined with the GTEx database. *p < 0.05, **p < 0.01, ***p < 0.001, and ****p < 0.0001.

**FIGURE 2:**
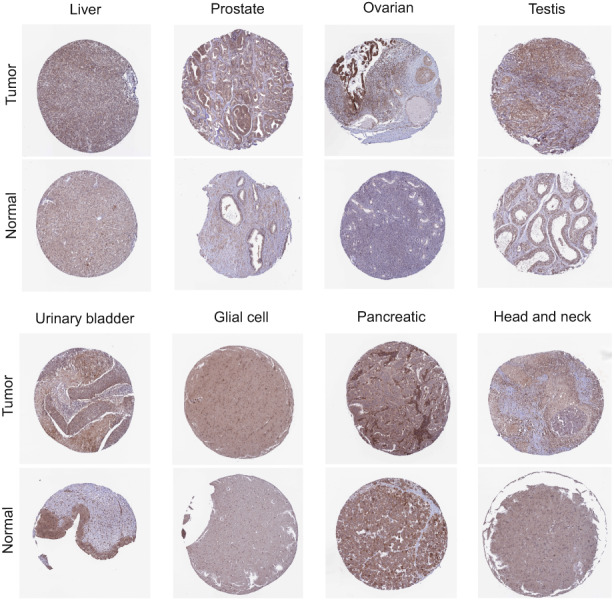
Immunohistochemistry staining of COMMD2 protein in normal and tumor tissues based on the HPA database.

We found that COMMD2 expression levels were high in various tumor cell lines based on the Cancer Cell Line Encyclopedia (CCLE) database (Figure S1). Then, we analyzed the differential expression of COMMD2 between the tumors with paired or non‐paired normal samples depending on the TCGA (Figure 1A) and SangerBox databases (Figure 1B), and it was shown that COMMD2 expression was markedly upregulated in BLCA, cholangiocarcinoma (CHOL), colon adenocarcinoma (COAD), esophageal carcinoma (ESCA), HNSC, LIHC, lung squamous cell carcinoma (LUSC), GBM, and stomach cancer (STAD), whereas it was downregulated in kidney renal clear cell carcinoma (KICH), lung adenocarcinoma (LUAD), and thyroid carcinoma (THCA). Furthermore, considering the small number of normal samples in TCGA, we combined TCGA with the GTEx database to further evaluate the differences in mRNA expression of COMMD2 in a broad manner in pan‐cancer. It was demonstrated that COMMD2 is highly expressed in most tumors in comparison to normal samples (Figure 1C). Finally, we performed immunohistochemistry from the HPA database to evaluate expression of COMMD2 at the protein level. Compared to normal tissues, COMMD2 protein was expressed at higher levels in LIHC, PRAD, TGCT, OV, BLCA, GBM, PAAD, and HNSC (Figure 2).

### 3.2 Survival analysis of COMMD2 in pan‐cancer

**FIGURE 3:**
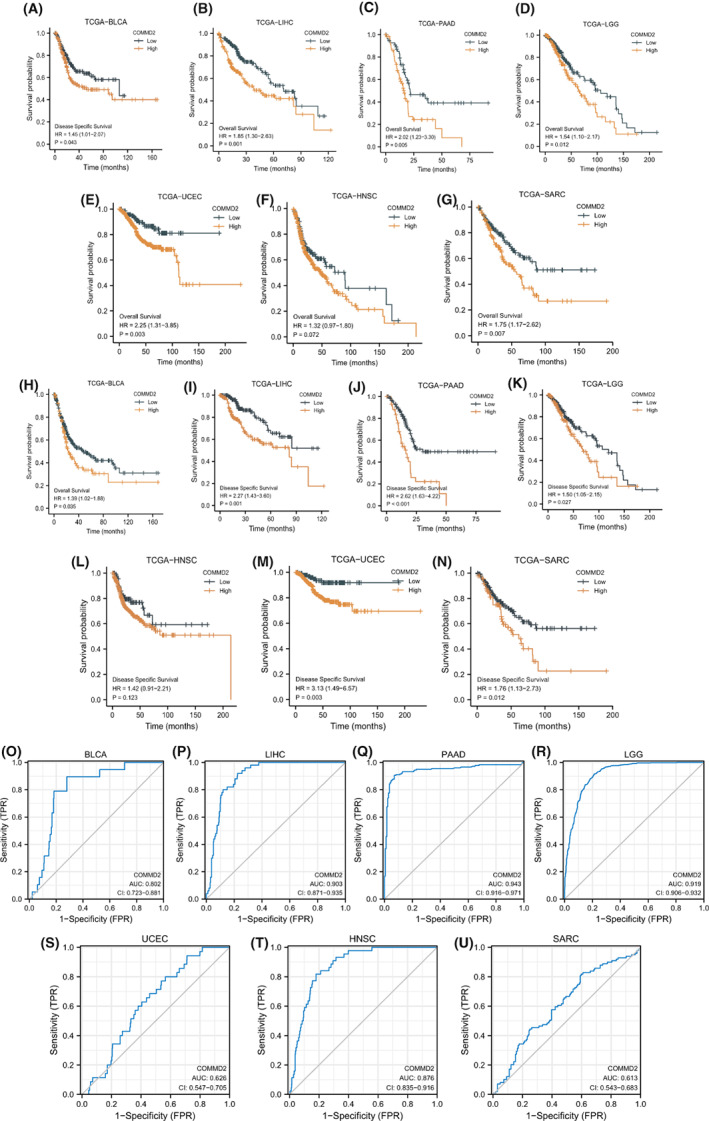
Survival analysis of COMMD2 in pan‐cancer. COMMD2 expression with OS survival curves of BLCA (A), LIHC (B), PAAD (C), LGG (D), UCEC (E), HNSC (F), and SARC (G). COMMD2 expression and survival curve of DSS in BLCA (H), LIHC (I), PAAD (J), LGG (K), UCEC (L), HNSC (M), and SARC (N). ROC curves for the relationship of COMMD2 expression and the malignant progression of BLCA (O), LIHC (P), PAAD (Q), LGG (R), UCEC (S), HNSC (T), and SARC (U).

To assess the correlation between COMMD2 gene expression and patient prognosis in different tumors, univariate Cox regression analysis and Kaplan–Meier analysis (KM analysis) of COMMD2 in pan‐cancer were performed relying on the TCGA dataset. The forest plots indicated that COMMD2 expression significantly correlated with OS in PAAD (*p* < 0.001), LGG (*p* < 0.001), LIHC (*p* < 0.001), ACC (*p* = 0.02), and BLCA (*p* = 0.05), while it was a gene of lower risk in KIRC (*p* < 0.001) (Figure S2A). Kaplan–Meier analysis (KM analysis) of COMMD2 in pan‐cancer was performed relying on the TCGA dataset. Kaplan–Meier survival curves displayed a tendency for higher COMMD2 expression toward shorter OS in patients with LIHC (*p* = 0.001), BLCA (*p* = 0.035), PAAD (*p* < 0.001), LGG (*p* = 0.012), UCEC (*p* = 0.003), and SARC (*p* = 0.006) (Figure 3A‐G). Moreover, the relationship between COMMD2 expression and DSS was determined by Cox regression analysis, which showed that enhanced COMMD2 expression had a negative impact on survival in patients with PAAD (*p* < 0.001), LIHC (*p* < 0.001), LGG (*p* < 0.001), ACC (*p* = 0.02), and LUAD (*p* = 0.03) (Figure S2B). Kaplan–Meier analysis also revealed the significant expression of COMMD2 in LIHC (*p* = 0.001), BLCA (*p* = 0.035), PAAD (*p* < 0.001), LGG (*p* = 0.012), UCEC (*p* = 0.003), and SARC (*p* = 0.006) (Figure 3H‐N), which had a worse effect on DSS. After that, we analyzed Receiver Operating Characteristic (ROC) curves to explore the effect of COMMD2 expression on the deterioration of various tumors. The area under the curve (AUC) values of the ROC curves predicted that the expression of COMMD2 might be related to the progression of BLCA, LIHC, LGG, PAAD, UCEC, HNSC, and SARC (Figure 3O‐U). Combining the results of the abovementioned analyses, we can speculate that COMMD2 expression may play a critical role in the survival of patients with tumors.

### 3.3 Correlation between COMMD2 expression and clinicopathological, immune, and molecular subtypes

**FIGURE 4:**
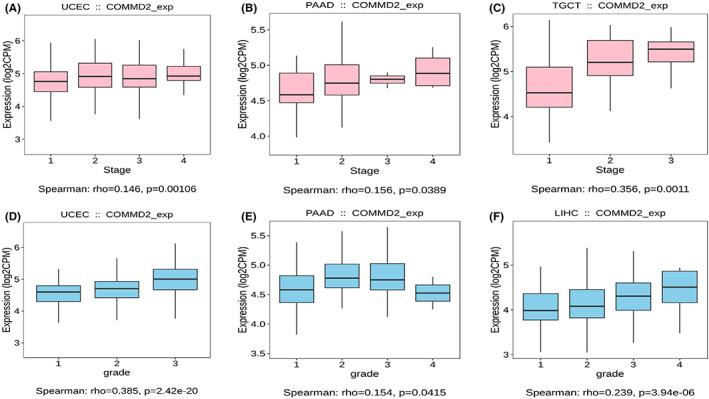
Relationship of COMMD2 expression and tumor stage and grade. COMMD2 expression and tumor stage in UCEC (A), PAAD (B), and TGCT (C). COMMD2 expression and tumor grade in UCEC (D), PAAD (E), and LIHC (F).

**FIGURE 5:**
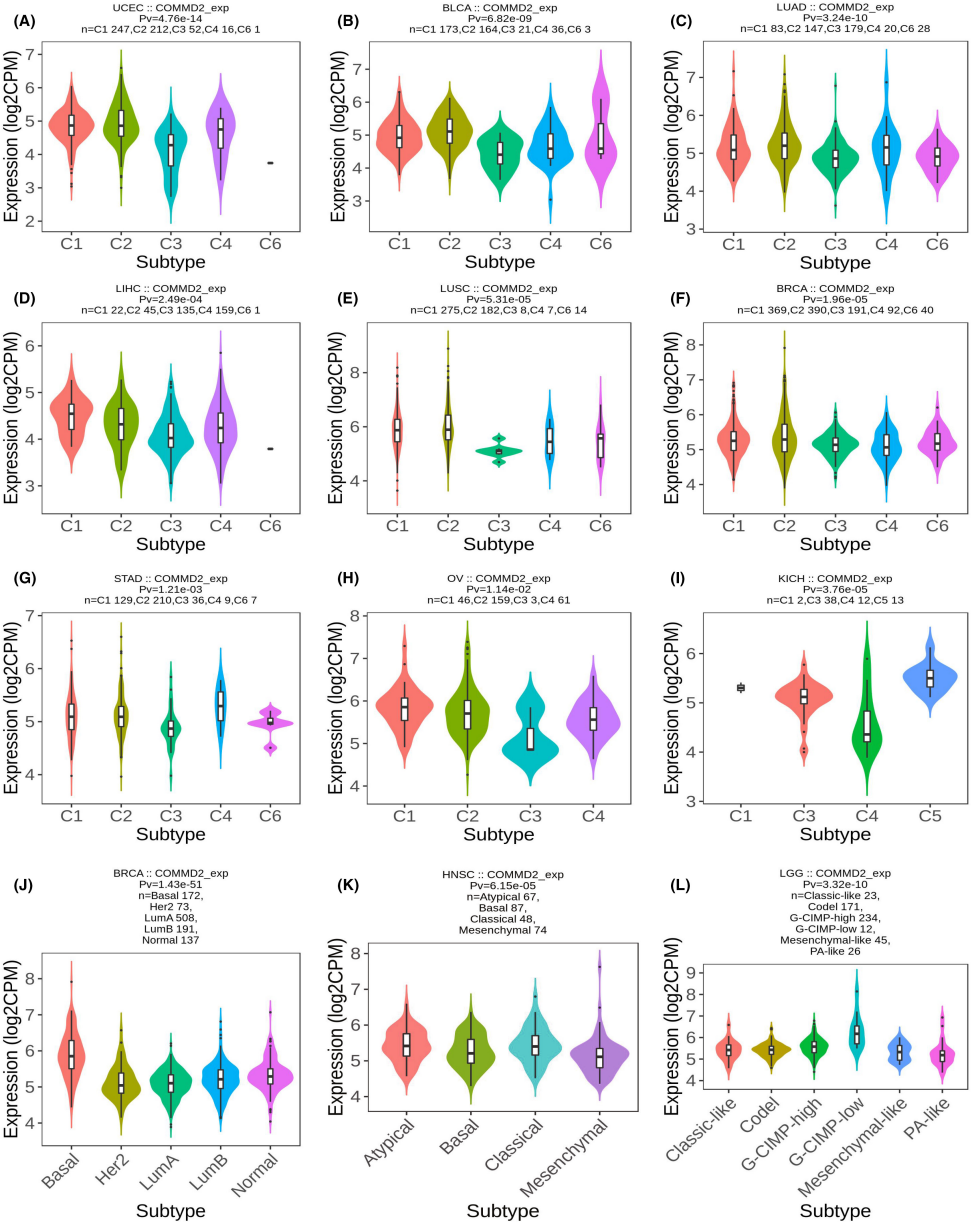
Relationship of COMMD2 expression with immune subtype in BLCA (A), UCEC (B), LUAD (C), LIHC (D), LUSC (E), BRCA (F), STAD (G), OV (H), and KICH (I). Association of COMMD2 expression with the molecular subtype in BRCA (G), HNSC (K), and LGG (L).

Increasing evidence demonstrates that different immunophenotypes can mirror comprehensively the overall immune status and microenvironment of tumors, which is intimately relevant to immunotherapy responses.^31^ Distinguishing molecular subtypes of cancer contributes to the choice of molecular targeted therapies and immunotherapy strategies.^32^ The TISIDB database was used to further illustrate the association of COMMD2 expression with the patient's clinicopathological, immune, and molecular subtypes. As shown in the graphic, we found that the expression levels of COMMD2 were more pronounced in higher stages or grades in UCEC, PAAD, TGCT, and LIHC (Figure 4A‐F). Our findings also revealed that COMMD2 expression varied significantly among different immune subtypes in UCEC, BLCA, LUAD, LIHC, LUSC, BRCA, STAD, OV, and KICH, with the lowest COMMD2 expression in the C3 type in the majority of tumors apart from KICH (C4) (Figure 5A‐I). Regarding diverse molecular subtypes, we noticed differences in several types of cancers, such as BRCA, HNSC, and LGG (Figure 5G‐L). Thus, it may be stated that COMMD2 may play a essential role in regulating the responsiveness of tumor immunotherapy and targeted therapy.

### 3.4 Methylation and genetic alteration analysis of COMMD2 in pan‐cancer

**FIGURE 6:**
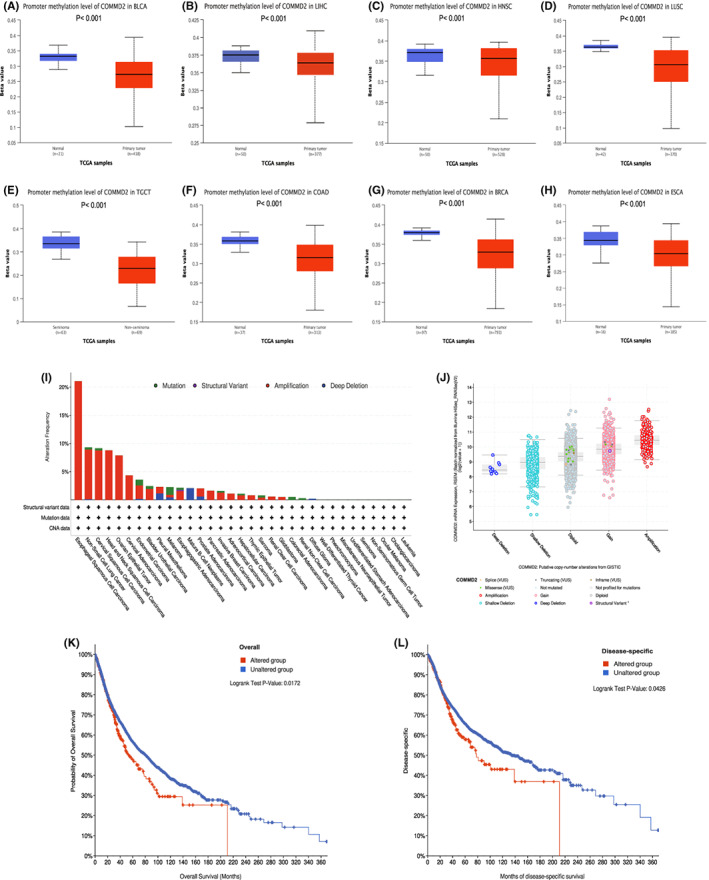
Methylation and genetic alterations of COMMD2 in pan‐cancer. (A) Methylation level of COMMD2 in BLCA (A), LIHC (B), HNSC (C), LUSC (D), TGCT (E), COAD (F), BRCA (G) and ESCA (H). (I) Mutation frequency of COMMD2 in different cancers. (J) Putative copy‐number alterations of COMMD2 from GISTI. (K) Genetic alterations of COMMD2 and OS. (L) Genetic alterations of COMMD2 and DSS.

Levels of promoter methylation of COMMD2 in eight types of tumors and normal tissues were analyzed using the UALCAN online platform based on TCGA samples. DNA methylation levels of COMMD2 were prominently higher in the tumor groups than in the normal cohorts (Figure 6A‐H). The cBioPortal database was used to explore the genetic alterations of COMMD2 in different tumors, as illustrated in Figure 6I, which ranked first in terms of the proportion of all genetic alteration types, particularly in esophageal squamous cell carcinoma, NSCLC, cervical squamous cell carcinoma, and HNSC. Another vital type of mutation is copy number modifications, the most prevalent of which are amplification and gain (Figure 6J). We found that COMMD2 mutations had an influence on patient survival, with the altered group's OS (*p* = 0.0172) and DSS (*p* = 0.0426) being statistically lower than those of the unaltered group (Figure 6K‐L).

### 3.5 Correlation of COMMD2 expression and tumor mutational burden, microsatellite instability, and stemness index

**FIGURE 7:**
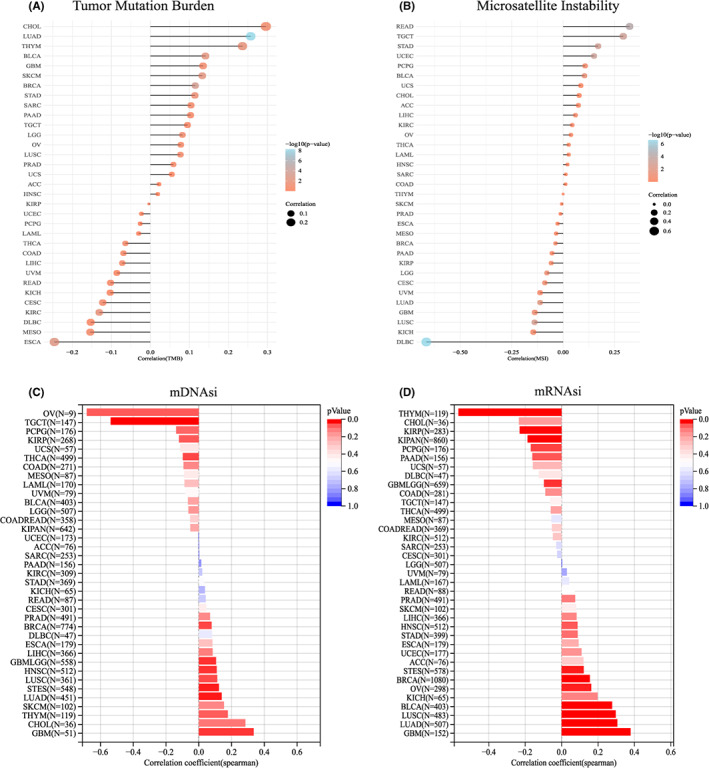
Relationship of COMMD2 expression and TMB (A), MSI (B), mDNAsi (C), and mRNAsi (D) in pan‐cancer.

TMB is a quantitative biomarker that reflects the sensitivity to immunotherapy.^33^ Tumor cells with a greater TMB are easier for the immune system to recognize, and immunotherapy is more likely to work for the patient. TMB was found to be favorably connected with COMMD2 expression in CHOL, LUAD, BLCA, and GBM, but negatively in case of ESCA, mesothelioma (MESO), and diffuse large B‐cell lymphoma (DLBC) (Figure 7A). MSI is mainly caused by the absence of DNA mismatch repair systems, and it can be used to forecast the prognosis of the tumor and the effectiveness of immunotherapy.^34^ In rectal adenocarcinoma (READ), TGCT, STAD, UCEC, and BLCA, COMMD2 expression was positively correlated with MSI; however, COMMD2 had an adverse relationship with MSI in DLBC, KICH, and LUSC (Figure 7B). The tumor stemness index is associated with tumor occurrence and metastasis, and a higher stemness index seems to be directly related to the degree of progression of multiple types of cancers.^35^ Gene expression‐based stemness index (mRNAsi) and DNA methylation‐based stemness index (mDNAsi) are important indices for evaluating tumor stemness. Based on COMMD2 expression and DNA methylation levels, we investigated the association between COMMD2 and the tumor stemness indices mRNAsi and mDNAsi. COMMD2 had a positive correlation with mRNAsi and mDNAsi in GBM, LUAD, LIHC, BRCA, PRAD, and HNSC, but negatively correlated with mRNAsi and mDNAsi in kidney renal papillary cell carcinoma (KIRP), COAD, THCA, and PCPG. Interestingly, COMMD2 was positively correlated with mRNAsi but negatively correlated with mDNAsi in BLCA and OV. This outcome could be the result of differences between mRNAsi and mDNAsi brought on by DNA hypermethylation. (Figure 7C‐D).

### 3.6 Analysis of COMMD2 expression with immune cells infiltration and the ESTIMATE score

**FIGURE 8:**
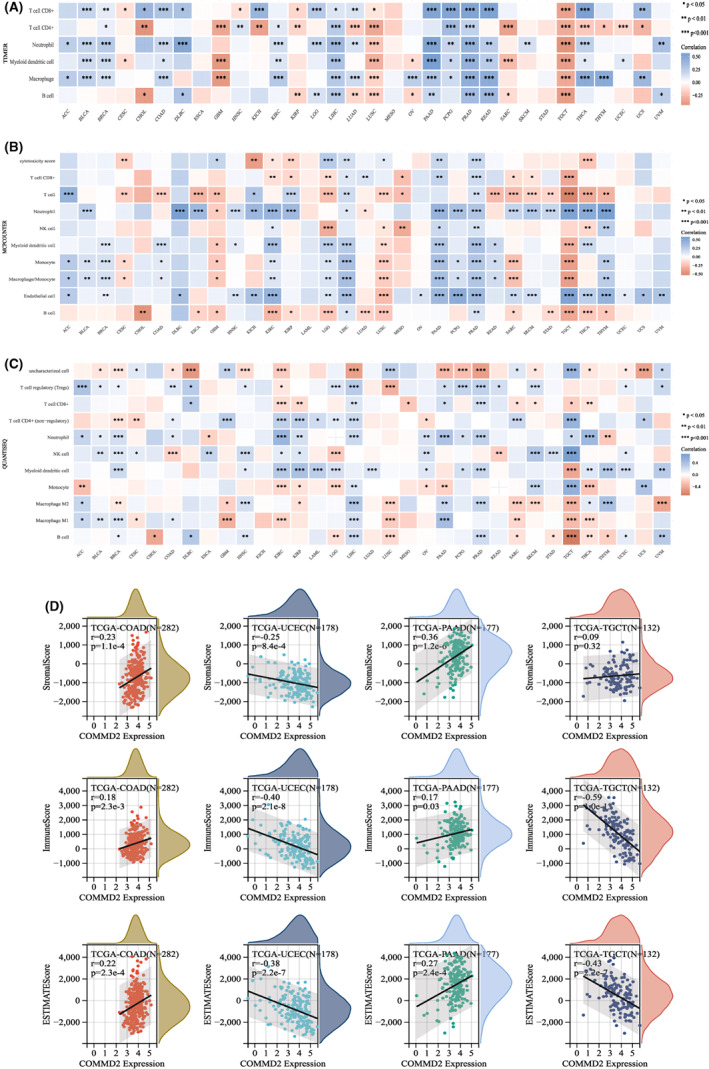
Association of COMMD2 expression and immune infiltration and ESTIMATE Score. The relationship between COMMD2 expression and immune cell infiltration evaluated via the TIMER algorithm (A), MCPCOUNTER algorithm (B), and QUANTISEQ algorithm(C). (D) COMMD2 expression and ESTIMATEScore, ImmuneScore and StromalScore in COAD, UCEC, PAAD, and TGCT. *p < 0.05, **p < 0.01, and ***p < 0.001.

The tumor microenvironment (TME) is important for the understanding and treatment of cancer. Understanding the dynamic functional interactions in this complex environment will help researchers develop more effective cancer‐fighting strategies.^36^ As one of the most essential components in the TME, tumor‐infiltrating immune cells (TIICs) have been a key origin of markers for predicting the survival and guiding immunotherapy for malignant tumor patients.^37^ For illustrating the relationship between COMMD2 and immune cell infiltration more comprehensively and accurately, various algorithms have been exploited to determine the relevance of COMMD2 expression and immune cell infiltration in diverse types of cancers. Based on the TIMER algorithm, COMMD2 expression was significantly correlated with six types of infiltrating immune cells, including CD8^+^ T cells, CD4^+^ T cells, neutrophils, dendritic cells, macrophages, and B cells, and in the majority of cancer types (Figure 8A), there was a positive association with PRAD and LIHC, whereas it was a negative correlation with TGCT and LUSC (*p < 0.05, **p < 0.01, ***p < 0.00). Furthermore, the MCPCOUNTER algorithm showed that neutrophil and endothelial cells were positively correlated with COMMD2 expression in most types of cancers. In contrast, B and T cell levels were negatively correlated with COMMD2 expression in most cases. PRAD and LIHC consistently correlated with most of tumor‐infiltrating immune cells (*p < 0.05, **p < 0.01, ***p < 0.001) (Figure 8B). The results of the QUANTISEQ algorithm also indicated that PRAD and LIHC were associated with the bulk of the TIICs (*p < 0.05, **p < 0.01, ***p < 0.001) (Figure 8C). The StromalScore, ImmuneScore, and EstimateScore were calculated in COAD, UCEC, PAAD, and TGCT (Figure 8D); COAD and PAAD had a statistically significant positive relationship with them, but UCEC and TGCT had the opposite result. The abovementioned analysis revealed that COMMD2 expression might be tightly linked to the extent of immune infiltration and immune responses in human tumors.

### 3.7 COMMD2 expression is correlated with immune checkpoint genes (ICGs) and drug sensitivity

**FIGURE 9:**
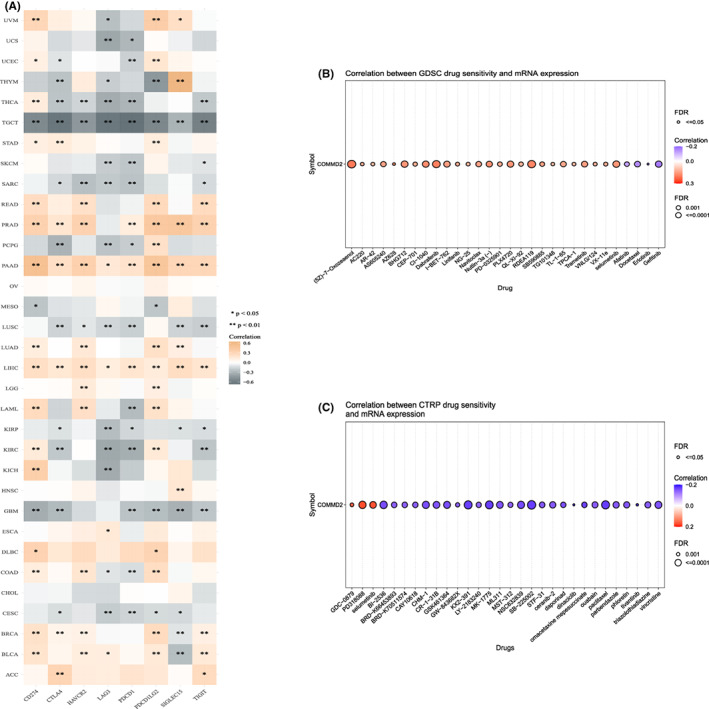
Analysis of the relevance between COMMD2 expression and immune checkpoint inhibitors and drug sensitivity. (A) Co‐expression of COMMD2 and immune checkpoint inhibitors. (B) GDSC drug sensitivity and COMMD2 mRNA expression. (C) CTRP drug sensitivity and COMMD2 mRNA expression. *p < 0.05, **p < 0.01, and ***p < 0.001.

ICGs are crucial in preventing self‐reactivity and constitute a novel target for tumor‐specific therapeutic strategies.^38^ We analyzed the expression profile of COMMD2 gene and ICGs (CD274, CTLA4, HAVCR2, LAG3, PDCD1, PDCD1LG2, SIGLEC15, and TIGIT) at a pan‐cancer level (Figure 9A). COMMD2 expression in READ, PRAD, PAAD, LUAD, LIHC, BRCA, and BLCA is consistent with most of the immune‐related genes; however, contrasting results were observed in THYM, THCA, TGCT, SARC, LUSC, KIRP, GBM, and CESC. The GSCA online platform was used to calculate the association between COMMD2 gene expression and sensitivities of the top 30 drugs, based on data from the GDSC and CTRP databases. GDSC data analysis revealed that the sensitivity to 26 chemicals was positively linked to COMMD2 mRNA expression, with a positive correlation with four chemicals (*p* ≤ 0.001) (Figure 9B). The CTRP database analysis revealed an adverse correlation between the sensitivity of 27 drugs and COMMD2 mRNA expression, while three drugs had an opposite relationship (*p* < 0.001) (Figure 9C).

### 3.8 Pathway analysis of COMMD2

**FIGURE 10:**
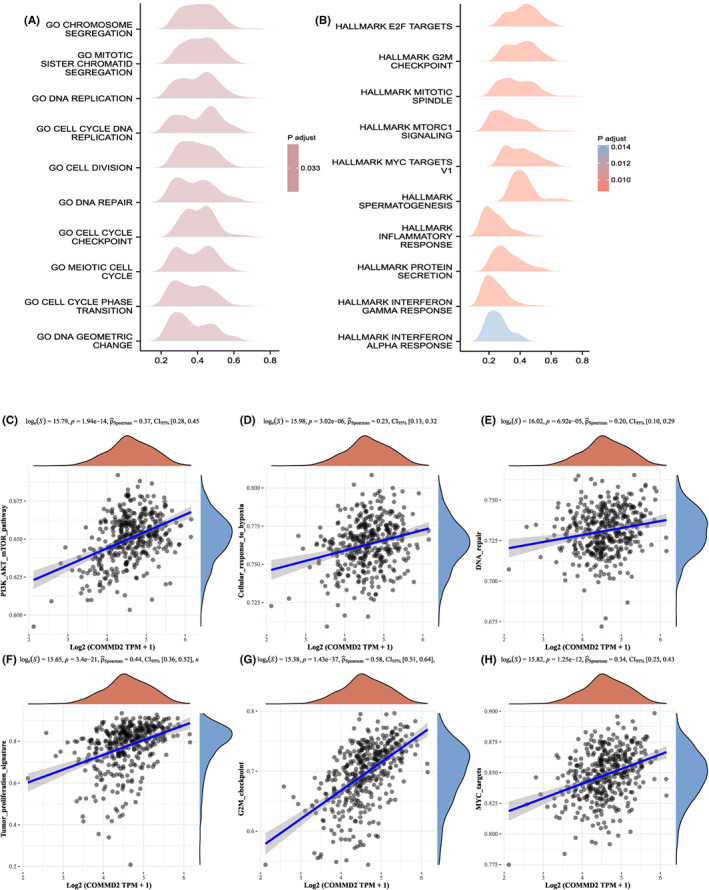
GSEA analysis and pathway analysis of COMMD2 in BLCA. Ridge plot of GSEA analysis respectively taken from the “c5.bp.v7.2.symbols.gmt” gene set (A) and “h.all.v7.2.symbols.gmt” gene set (B) as the references in BLCA. (C‐H) Association of COMMD2 and pathways related with tumor in BLCA.

Since we have already found that the expression level of COMMD2 affects the prognosis and treatment of tumor patients, we would like to investigate the potential molecular biological processes of COMMD2. First, a gene–gene interaction network was built to identify genes with COMMD2‐related functions. Based on the results, the top 20 genes related to COMMD2 were identified (Figure S3), which included the COMMD family, CCDC22, CCDC93, and SCLT1. Moreover, to investigate the potential biological processes by which COMMD2 influences the occurrence and development of tumors, GSEA analysis was performed in BLCA. Taking “c5.bp.v7.2.symbols.gmt” as the reference gene set, the genes which were enriched were primarily related to chromosome segregation, mitotic sister chromatid segregation, DNA replication, and cell cycle DNA replication related to the G0 stage in BLCA (Figure 10A). Next, we ran an enrichment analysis of HALLMARK pathways depending on the “h.all.v7.2.symbols.gmt” gene set. The results demonstrated in the mountain map indicated that genes which co‐expressed with COMMD2 were significantly enriched in hallmark E2F targets, hallmark mitotic spindle, hallmark G2M checkpoint, and hallmark MTORC1 signaling in BLCA (Figure 10B). Based on the abovementioned results, we speculated that COMMD2 might have a prominent influence in regulating the cell cycle of tumor cells. Furthermore, we determined the correlation between COMMD2 expression and the activity of various signaling pathways. In BLCA, COMMD2 activated tumor‐related pathways, such as PI3K/AKT/mTOR, cellular response to hypoxia, DNA repair, tumor proliferation signature, G2M checkpoint, and MYC targets (Figure 10C‐H). Hence, these results strongly suggest that COMMD2 may have a vital effect on bladder tumorigenesis and development.

### 3.9 COMMD2 boosts in vitro proliferation and migration of BLCA cells

**FIGURE 11:**
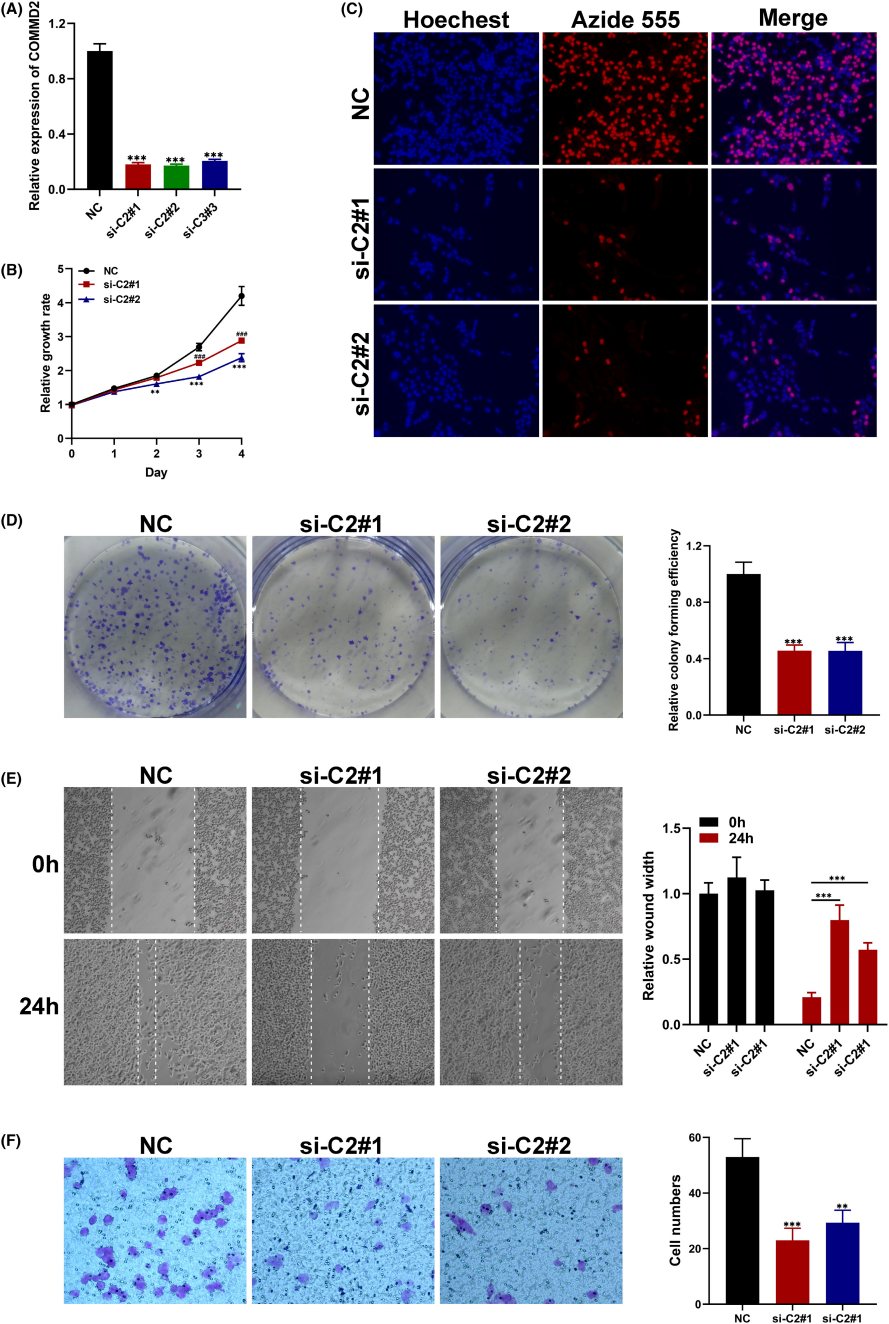
COMMD2's biological roles in bladder cancer and uterine corpus endometrial carcinoma cells. (A) In UMUC3 and HEC‐1‐A, verification of COMMD2 knockdown efficiency. CCK‐8 (B), EdU assay (C), colony formation assay (D), wound healing (E), and transwell assay (F) were performed to confirm the biological function of COMMD2 in bladder cancer and uterine corpus endometrial carcinoma cells. **p < 0.01, ***p < 0.001, and ###*p* < 0.001.

We performed qRT‐PCR to assess COMMD2 expression in BLCA and UCEC cells. The expression levels of COMMD2 in UMUC3 and HEC‐1‐A cells were knocked down using specific COMMD2‐targeting siRNAs (Figure 11A; Figure S4A). The CCK‐8, EdU, wound healing, and transwell assays revealed that knocking down COMMD2 stopped UMUC3 and HEC‐1‐A cells from proliferating and migrating (Figure 11B‐F; Figure S4B‐E). Moreover, we validated that the expression of COMMD2 was upregulated in BLCA and UCEC tumor tissues compared with the normal tissue by performing IHC (Figure S5). Thus, these findings revealed that COMMD2 enhances the proliferation and migration of BLCA and UCEC cells in vitro.

## 4 DISCUSSION

Humans suffer horribly from tumors.^39^ Although different cancer treatments are available, the prognosis for multiple tumors remains poor. It is possible to gain a better understanding of cancer development and progression by identifying genes significantly associated with tumor treatment and prognosis. This study highlighted the value of COMMD2 in human tumors and demonstrated that COMMD2 is a promising prognostic biomarker and therapeutic target in pan‐ cancer.

Accumulating evidence suggests that the aberrant expression of the COMMD protein family acts in tumorigenesis and progression. NF‐κB is a classic inflammatory pathway protein that facilitates human tumors, COMMD1 can mediate the degradation of NF‐κB through ubiquitination to regulate NF‐κB expression negatively, and low expression of COMMD1 can boost cell proliferation and aggressive viability in prostate cancer,^40^ lung cancer,^41^ and neuroblastoma.^42^ COMMD10 is modestly expressed in liver cancer and can disrupt the NF‐κB signaling pathway, reduce proliferation, increase apoptosis, and improve the predictive value of BCLC staging.^26^ HIF‐1 regulates a number of target genes involved in hypoxia adaptability, inflammatory development, and tumor growth. A recent study found that COMMD10 inhibits the HIF1/CP ring and alters the Cu–Fe balance in HCC to increase iron mortality and radiation sensitivity.^43^ Overexpression of COMMD3 and COMMD7 contributed to HCC cell migration, invasion, and angiogenesis.^15^, ^44^ COMMD2 may take part in the progression of liver cancers and interact with the epithelial sodium channel (ENaC) to regulate sodium homeostasis and blood pressure.^3^, ^8^ Our bioinformatics analysis revealed that COMMD2 is upregulated in BLCA, CESC, CHOL, COAD, ESCA, GBM, HNSC, LIHC, LUSC, and STAD, which can predict worse OS and DSS. Moreover, COMMD2 expression is linked to tumor stage, immunotype, and molecular typing, which is in line with previous research on liver cancer.^3^

The essence of carcinogenesis occurs at the genomic level, where epigenetic modifications have a substantial impact on tumor heterogeneity, as well as on the tumor's diagnosis and treatment strategy.^45^, ^46^ Therefore, we examined COMMD2 gene's promoter methylation and gene variant status. DNA methylation levels of COMMD2 were lower in BLCA, LIHC, HNSC, LUSC, TGCT, COAD, BRCA, and ESCA tissues than in the normal tissues. COMMD2 has amplification mutations in most malignancies, especially ESCA, with mutation rates of more than 20%, and its mutations are also strongly linked to patient prognosis.

Immunotherapy is among the most major approaches in cancer treatment currently. The tumor mutation load is associated with immunotherapy responses in different cancer types.^47^ Patients with advanced gastrointestinal cancer with microsatellite high instability (MSI‐H) benefit from immune checkpoint inhibitors (ICIs).^48^ Hyper‐stem tumor cells express much lower levels of the main histocompatibility complex II but more significantly express cytokine members than low‐stem malignant cells, indicating immune evasion and interaction with surrounding immune cells in case of hyper‐stem malignant cells.^49^ In our study, COMMD2 expression was found to be tightly linked to TMB, MSI, mDNAsi, and mRNAsi in several tumors. In addition, COMMD2 expression was positively correlated with CD8^+^ T cells, macrophages, myeloid dendritic cells, and neutrophil‐infiltrated cells in numerous cancers, implying that COMMD2 had a impact on immune cell infiltration and may affect tumor immunotherapy.

In the previous analysis, we discovered that elevated COMMD2 gene expression in BLCA affects the prognosis of patients, and earlier research has indicated that copper metabolism is crucial in BLCA.^50^, ^51^ Given the irreplaceable function of the COMMD family in copper metabolism, we next examined whether COMMD2‐related pathways can be involved in BLCA and found that COMMD2 is intimately associated with DNA replication and the cell cycle in BLCA. In addition, we performed functional experiments in BLCA cell lines and UCEC cell lines and found COMMD2 facilitates the proliferation and migration of BLCA cells and UCEC cells. The mechanism by which COMMD2 promotes tumor progression needs to be clarified further, including how COMMD2 influences human tumor occurrence and progression.

## 5 CONCLUSION

Overall, this study offers comprehensive insight into the function of COMMD2 in human tumors and reveals that COMMD2 may serve as a useful prognostic biomarker and therapeutic target in pan‐cancer.

## AUTHOR CONTRIBUTIONS

**Panpan Tai:** Data curation (equal); investigation (equal). **Zhanwang Wang:** Formal analysis (equal); investigation (equal). **Xinyu Chen:** Conceptualization (equal); formal analysis (equal). **Aiyan Chen:** Methodology (equal). **Lian Gong:** Investigation (equal). **Yaxin cheng:** Formal analysis (equal). **Ke Cao:** Funding acquisition (equal); project administration (equal).

## FUNDING INFORMATION

This study was supported by the National Natural Science Foundation of China (81874137), the science and technology innovation Program of Hunan Province (2020RC4011), the Hunan Province Science and Technology Talent Promotion Project (2019TJ‐Q10), Young Scholars of “Furong Scholar Program” in Hunan Province, and the Wisdom Accumulation and Talent Cultivation Project of the Third Xiangya hospital of Central South University (BJ202001).

## CONFLICT OF INTERESTS

The authors declared no conflict of interest.

## Supporting information


Figure S1

Figure S2

Figure S3

Figure S4

Figure S5

Table S1

## Data Availability

The data presented in the study are available upon reasonable request to the corresponding author.
