# Supplementary figures and images for: Multi‐omics analysis of the oncogenic value of copper Metabolism‐Related protein COMMD2 in human cancers
Source: Cancer Med. 2022 Oct 7;12(10):11941–59. doi: 10.1002/cam4.5320 (PMC10242316; doi:10.1002/cam4.5320)

Supplementary Figure 1

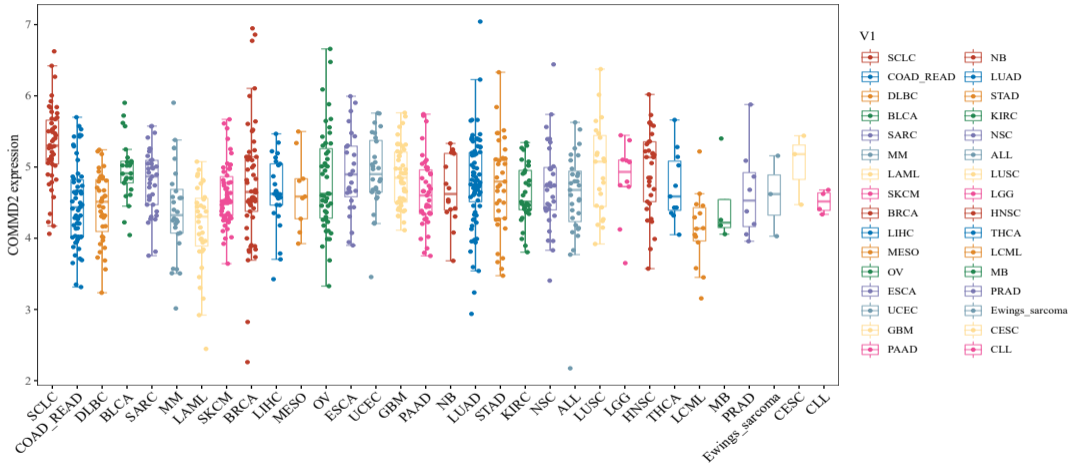

Supplement: Supplementary file 1 — Figure S1 [file CAM4-12-11941-s004.pdf]

# Supplementary Figure 2

A

## Overall Survival

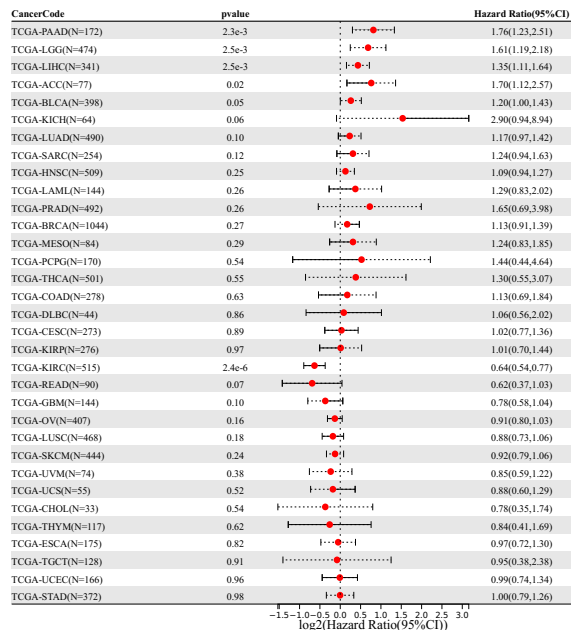

B

## Disease Specific Survival

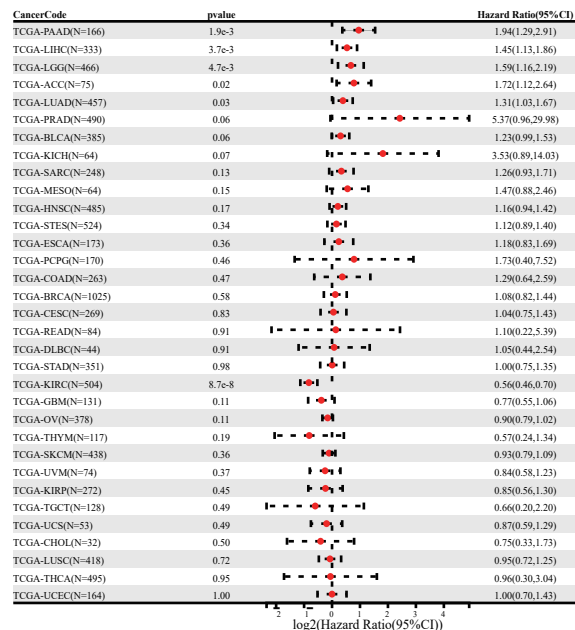

Supplement: Supplementary file 2 — Figure S2 [file CAM4-12-11941-s001.pdf]

Supplementary Figure 3

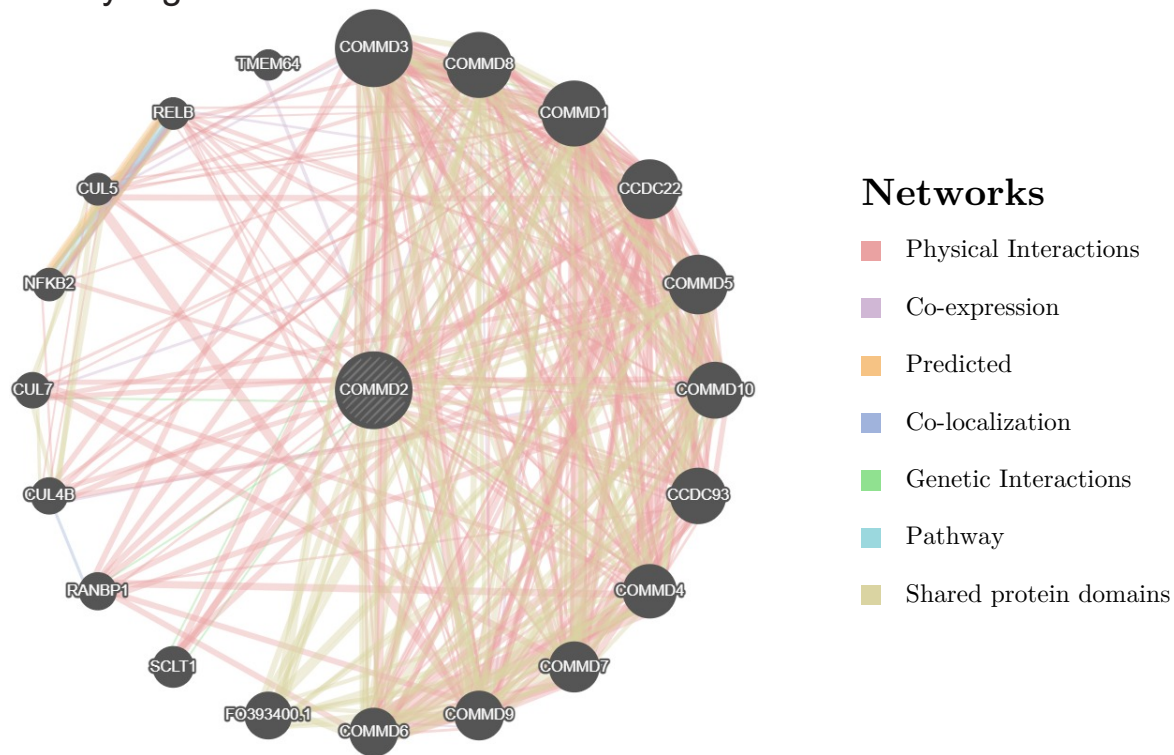

Supplement: Supplementary file 3 — Figure S3 [file CAM4-12-11941-s005.pdf]

**A**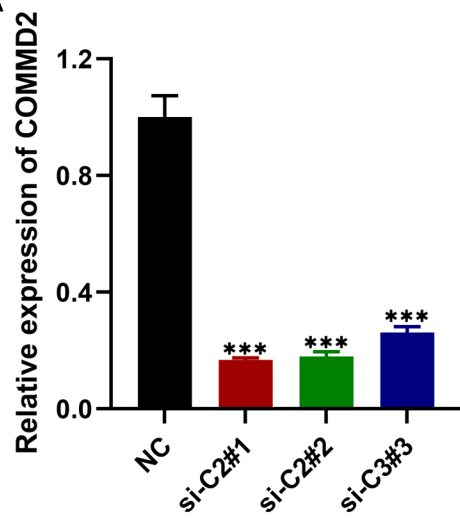**B**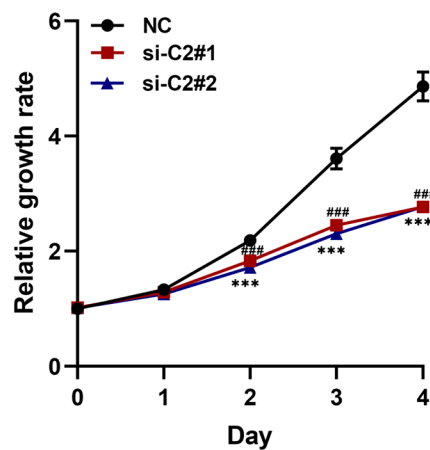**C**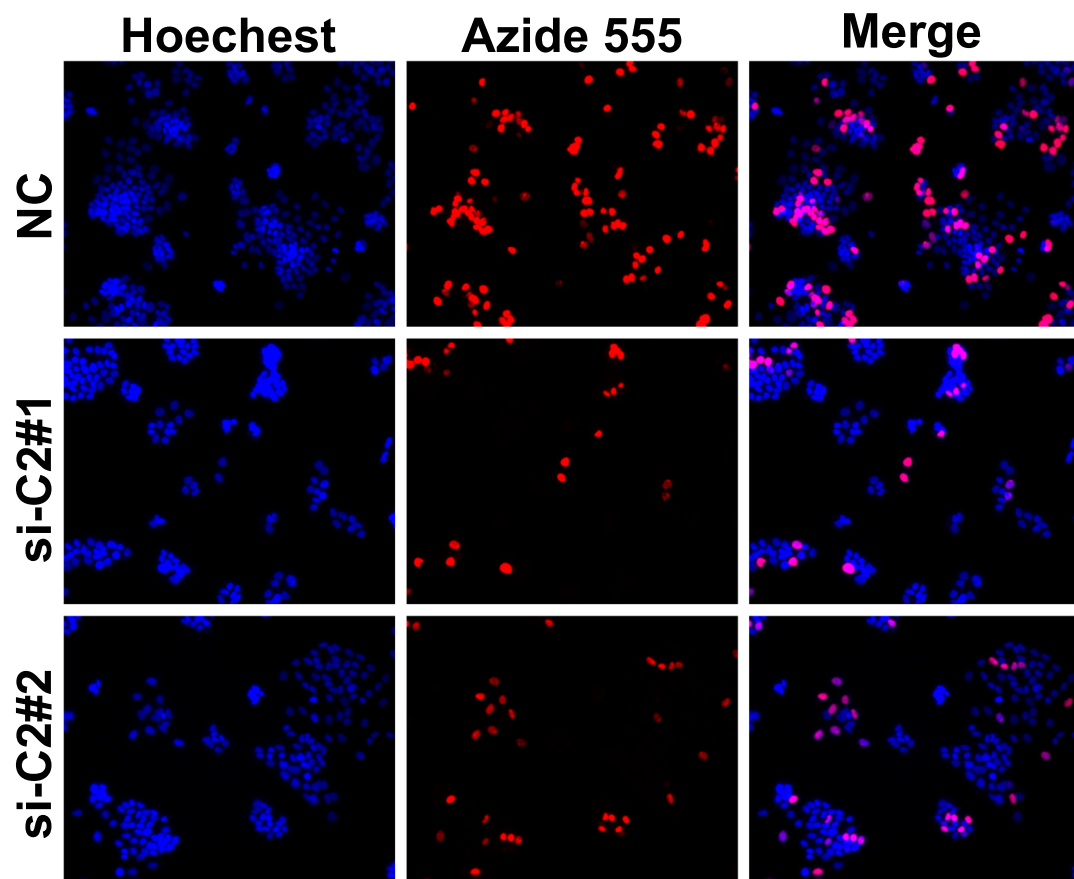**D**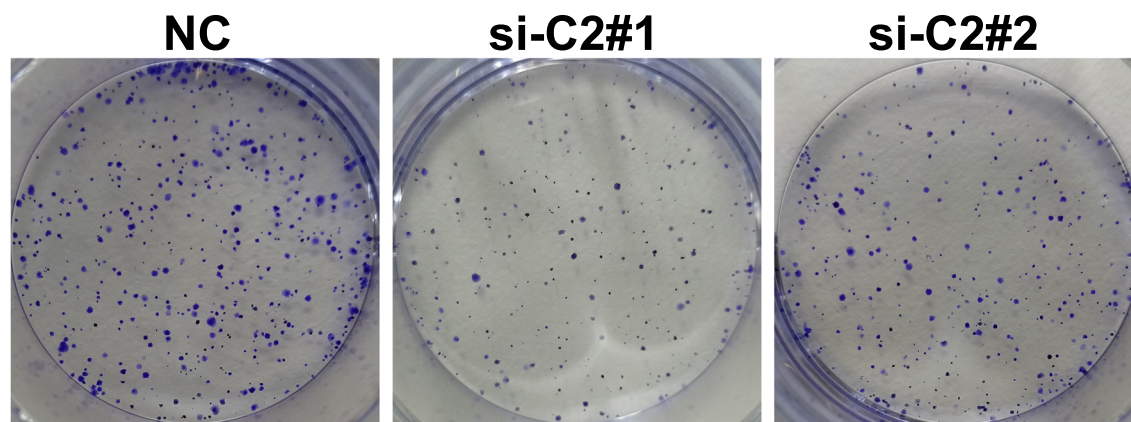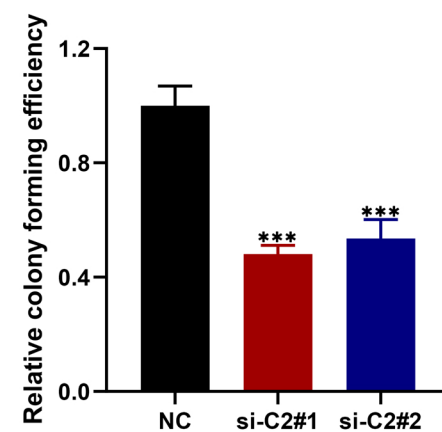**E**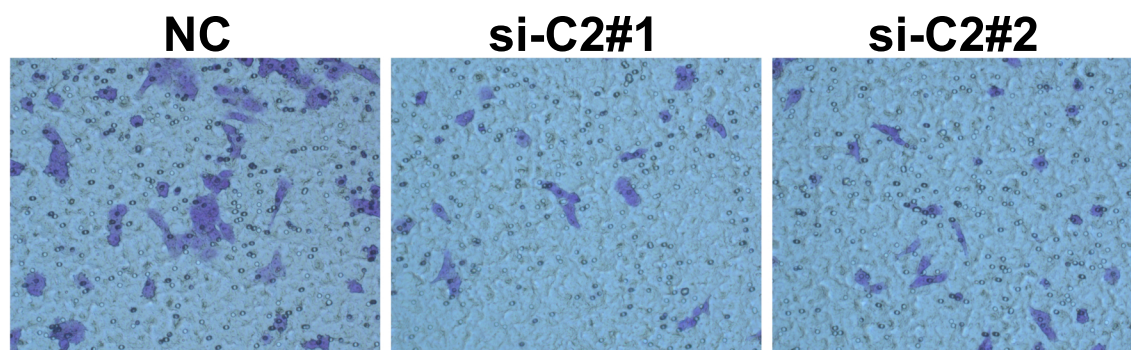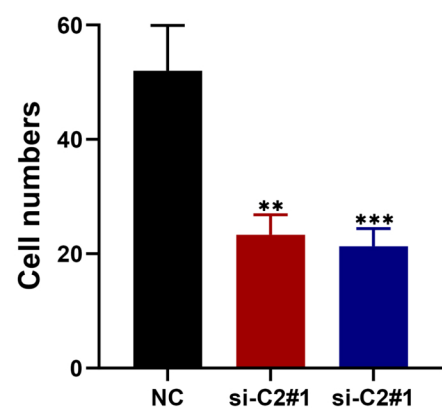

Supplement: Supplementary file 4 — Figure S4 [file CAM4-12-11941-s006.pdf]
