# Supplementary material for: Multi‐omics analysis of the oncogenic value of copper Metabolism‐Related protein COMMD2 in human cancers
Source: Cancer Med. 2022 Oct 7;12(10):11941–59. doi: 10.1002/cam4.5320 (PMC10242316; doi:10.1002/cam4.5320)
Supplement: Supplementary file 5 — Figure S5 [file CAM4-12-11941-s003.pdf]

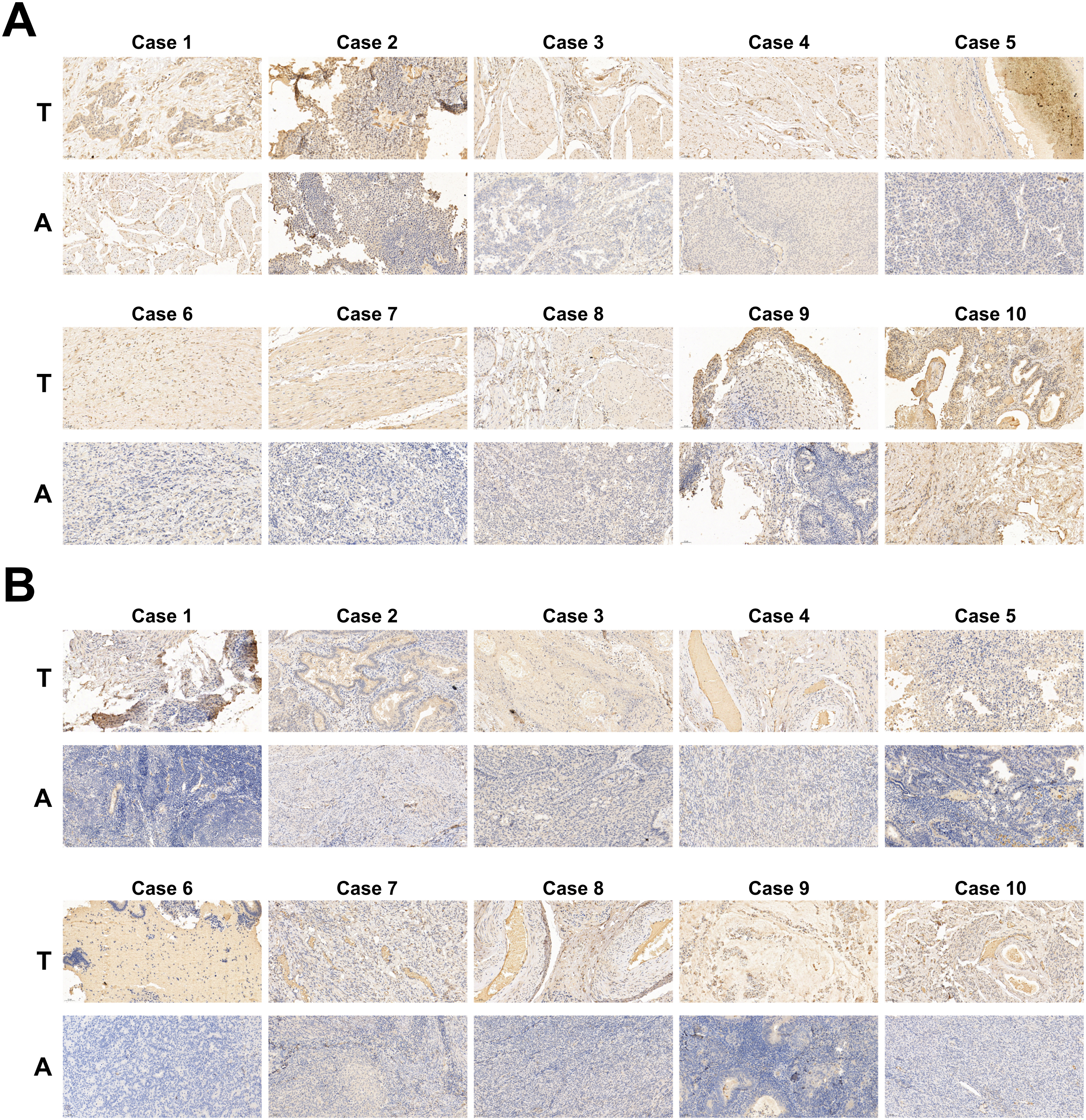
A
Case 1
Case 2
Case 3
Case 4
Case 5
T
A
Case 6
Case 7
Case 8
Case 9
Case 10
T
A
B
Case 1
Case 2
Case 3
Case 4
Case 5
T
A
Case 6
Case 7
Case 8
Case 9
Case 10
T
A
