# Supplementary material for: Multi‐omics analysis of the oncogenic value of copper Metabolism‐Related protein COMMD2 in human cancers
Source: Cancer Med. 2022 Oct 7;12(10):11941–59. doi: 10.1002/cam4.5320 (PMC10242316; doi:10.1002/cam4.5320)
Supplement: Supplementary file 6 — Table S1 [file CAM4-12-11941-s002.docx]

| **Sequences of Primers** | |
| --- | --- |
| **COMMD2** | **Forward:** GCAAGTAGAAGTCTCAG GCAAC |
|  | **Reverse:** TGCGAACAACTCTCCTACAGT |
| **GAPDH** | **Forward:** GTCAGCCGCATCTT CTTT |
|  | **Reverse:** CGCCCAATACGACCAAAT |
| **Sequences of siRNAs** | |
| **si-C2#1** | **Forward:** GGAAUUGUCCGAGGAGCAUTT |
|  | **Reverse:** AUGCUCCUCGGACAAUUCCTT |
| **si-C2#2** | **Forward:** GGCGGAUUGCUGUGGAAUUTT |
|  | **Reverse:** AAUUCCACAGCAAUCCGCCTT |
| **si-C2#3** | **Forward:** GCAUGGUGUGGAAGGAUUATT |
|  | **Reverse:** UAAUCCUUCCACACCAUGCTT |
| **si-NC** | **Forward:** UUCUCCGAACGUGUCACGUTT |
|  | **Reverse:** ACGUGACACGUUCGGAGAATT |
